# Health-related quality of life after pediatric traumatic brain injury: A qualitative comparison between children’s and parents’ perspectives

**DOI:** 10.1371/journal.pone.0246514

**Published:** 2021-02-10

**Authors:** Ugne Krenz, Dagmar Timmermann, Anastasia Gorbunova, Michael Lendt, Silke Schmidt, Nicole von Steinbuechel

**Affiliations:** 1 Department of Medical Psychology and Medical Sociology, Georg-August University, Göttingen, Germany; 2 Neuropediatrics, St. Mauritius Therapeutic Clinic, Meerbusch, Germany; 3 Department Health and Prevention, University of Greifswald, Greifswald, Germany; Charite Universitatsmedizin Berlin, GERMANY

## Abstract

**Background:**

Pediatric traumatic brain injury (TBI) may cause a wide range of symptoms, which can negatively affect the quality of life of patients and their entire families. No internationally and simultaneously developed disease-specific instrument exists for assessing pediatric health-related quality of life (HRQoL) after TBI. The aim of the current project is to provide original material from small group interviews with individuals after TBI concerning what they state is relevant for their HRQoL. This material is required for a further study to generate items for age-adapted questionnaires assessing the TBI-specific HRQoL of children and adolescents (C&A): the QOLIBRI-Kiddy/Kid/Ado and proxy versions (Quality of Life after Brain Injury–Kiddy/Kids/Adolescents/Proxy) for individuals aged 6–17 and their parents.

**Methods:**

The semi-structured interviews were conducted with separate small groups of C&A (n = 19), divided into three age groups (5-7y, 8-12y, 13-17y), after mild, moderate, and severe TBI, and with groups of the corresponding parents (n = 26). All interviews lasted for about 60 minutes, were recorded and transcribed verbatim. The statements were investigated by qualitative analyses and sorted into categories relevant to the HRQoL of C&A after TBI. Only descriptive group comparisons but no pairwise comparisons between children and corresponding parents were performed.

**Results:**

The analyses led to 32 subcategories, which were assigned to six main theoretically based HRQoL categories. Many agreements exist between the C&A’s and parents’ perspectives within the main categories, however their focus on HRQoL differs, especially concerning age-related contents. Parents of the youngest participant group already focus on topics such as autonomy, whereas this only becomes relevant for C&A from the age of eight years on. Interestingly, even 5-year-old children were able to discuss their HRQoL, which indicates the importance of a self-report instrument.

**Conclusions:**

Results obtained from this qualitative study identify the content of the HRQoL dimensions important for C&A after TBI and their parents. Both, differences and similarities in the children’s and the parents’ views were investigated, to get a first insight in valid dimensions for the prospective questionnaires to be developed. In a future study, items for the questionnaires will be deducted from the small group interview material and psychometrically tested in C&A after TBI from Germany. This study will address whether all statements were assigned to the suitable dimensions and whether differences between C&A and parents persist.

## Introduction

Traumatic brain injury (TBI) is a common issue in the pediatric population and affects children worldwide. It can cause a series of different sequelae with potentially significant effects on the patients’ and their families’ lives and thus on aspects of their health-related quality of life (HRQoL). Long-term consequences can relate to cognitive, emotional, behavioral and/or social areas [1–3]. With an incidence rate of 581 persons per 100,000 inhabitants, TBI represents the most frequent form of injury in children and adolescents (C&A) up to the age of 16 years in Germany [4, 5]. Moreover, TBI is the leading cause of morbidity and mortality among C&A in Europe and the US [6].

In 1948 the World Health Organization defined health as a “… state of complete physical, mental and social well-being and not merely the absence of disease and infirmity” [7]. In the late 1970s, Engel [8] introduced the biopsychosocial model into medical care and research, which increased interest in the effects of disease and treatment beyond physical outcomes. HRQoL, as a multidimensional construct, reflects these conceptions of subjective health. In recent years, the improvement of quality of life has become an important and accepted outcome criterion for health-related issues [9–11]. According to a definition of this construct by von Steinbuechel et al. [12, 13] HRQoL encompasses physical, emotional, cognitive, social and everyday aspects of subjective functioning and subjective well-being, which should preferably be self-rated by the individuals concerned.

HRQoL can be measured across all diseases and health conditions (generic) or else disease-specifically. Generic instruments provide an overview of the perceived health, health behavior, and subjective well-being of the person concerned, and allow different diseases and conditions to be compared. However, many studies emphasize the importance of using disease-specific tools because these are usually more sensitive to individual disease patterns [14–16]. Most importantly, they allow the core aspects to be identified that are most relevant to enhancing the HRQoL of individuals affected by a certain disease. TBI can negatively affect many different areas of HRQoL such as cognition and educational competences (e.g. executive functions, language, attention, processing speed), emotions (e.g. reduced self-esteem), social life (e.g. reduced social awareness), and lead to serious changes in lifestyle (e.g. reduced independence) [1]. Thus, the wellbeing of C&A impacted by these consequences can be addressed in more detail by administering a TBI-specific HRQoL instrument.

A disease-specific instrument does not yet exist for pediatric TBI populations. In the field of adult TBI, the disease-specific instrument Quality of Life after Traumatic Brain Injury (QOLIBRI–von Steinbuechel et al., [17, 18]) has been internationally established in various settings, including methodological and clinical research, clinical care, and rehabilitation. The tool comprises six dimensions of HRQoL after TBI: cognition, self, autonomy and daily life, social relationships, emotions and physical problems. The content, importance and wording of items describing these different HRQoL dimensions for adults may differ from those for children; thus the objective of this study was to provide original material systematized for three different age groups (6–7, 8–12 and 13–17 years of age), to later develop three different QOLIBRI-Kiddy/Kid/Ado questionnaires.

Coghill, Danckaerts, Sonuga-Barke, Sergeant and the ADHD European Guidelines Group [19] consider assessing information from the patient’s perspective to be a key element when determining relevant dimensions of HRQoL. Qualitative approaches (e.g. interviews in small groups) are appropriate ways of gathering insights into the specificity of experiences C&A make after TBI [20].

As already mentioned, subjective HRQoL should be assessed by patients themselves whenever possible. However, children after TBI may be physically and cognitively too impaired or too young to report reliably on their HRQoL. In such cases, parents could be interviewed with respect to how they believe their children might judge their respective HRQoL. Many researchers share the conviction that parents can validly evaluate the subjective HRQoL of their children [21–23]. However, some researchers report that parents often rate external aspects more accurately than internal ones [24, 25] and underestimate the quality of life of their children [26]. Likewise, in a systematic literature review by Upton, Lawford and Eiser [27] differences in parent-child agreement were found between domains of HRQoL for four different measures.

The results of more current studies differ with regard to the possible influence of the children’s and adolescents’ age on self- and proxy-agreement. For example, Gothwal, Bharani, and Mandal [28] report that the differences between children’s and parents’ reports on a child’s HRQoL are significantly larger for younger children with congenital glaucoma (8–12 years) than for the respective adolescents. Results of a longitudinal study by Rajmil, López-Aguilà, and Alonso [29], on the other hand, found that the degree of parent-child agreement in a general population sample decreased with the age of the children. Both studies assessed self and parent reports on the child’s HRQoL using the Kidscreen-27 questionnaire.

Here, we will report on the initial stage of the development of the QOLIBRI-Kiddy/Kid/Ado for C&A after TBI and corresponding proxy versions. First, the statements derived from small group interviews through qualitative analyses and their sorting into a category system will be described. For this purpose, group interviews in pediatric care constitute a valuable tool to gain insight into the subjective perspectives of C&A [30]. Some other studies already successfully used group interviews with a small sample of C&A for item-generation [e.g. 31, 32]. The participation of C&A after TBI and their parents are relevant as they enable the exploration of topics and wording important to them. Furthermore, the fact that no international disease-specific instrument exists to assess pediatric HRQoL after TBI, and in view of the inconsistent results from earlier studies on child-parent-agreement, we will investigate in a first exploratory inspection whether and to what extent the perceptions of HRQoL after pediatric TBI coincide or differ between the C&A after TBI and their parents. Despite of the exploratory character valuable information for the development of the questionnaires can be provided.

## Participants and methods

This study was approved by the ethical committee of the University Medical Center Goettingen. Written conformed consent was obtained from all individual participants involved in the study after they were told about the investigational nature of the study.

### Design

We conducted a qualitative approach in form of semi-structured interviews to investigate relevant aspects of HRQoL important to C&A after TBI. The methodological implementation of the interviews was based on the concept of focus groups [33–36] and the interviews were conducted based on the problem-centered interview method according to Witzel and Reiter [37]. The combination of both concepts and the use of an interview guideline ensured the comparability of the interviews.

### Recruitment of participants

After approval by the ethics committee (21/3/14), participants were recruited in a consecutive fashion from the medical files of four acute or rehabilitation clinics in Germany (Frankfurt, Goettingen, Greifswald, and Meerbusch). Patients were recruited by mail and given detailed information about the study. Everybody who consented to participate until four weeks after having been addressed, and who corresponded to the inclusion and exclusion criteria was included in our study. The inclusion criteria for the TBI group were an ICD-10 diagnosis of head injury (S06.0- S06.9) specifying the severity, at least 3 months but no more than 10 years post TBI, 5 to 17 years of age at the time of the study, and the ability to understand and answer questions. Consent to participation and to audio-recording had to be given both by the legal guardian and by the respective child or adolescent. Participants were attributed to the interview groups concerning the criteria of age, TBI severity and gender in order to obtain the broadest possible spectrum of perspectives from C&A and their parents. Each C&A received a little toy or a small book voucher as a token of our appreciation, as well as a travel allowance.

Additionally, interviews were conducted with healthy C&A and their parents in order to investigate whether HRQoL beliefs do indeed differ between these groups. Since this study focuses on the parent-child agreement concerning HRQoL after TBI, no data on these participants will be presented here.

### Procedure

Eight semi-structured group interviews were conducted with 20 C&A (participants per interview: *M* = 3, *R* = 1–5) and six were conducted with their parents (*n* = 26; participants per interview: *M* = 4, *R* = 1–7). One interview with a child and one of its parents was conducted as an individual interview. One participant was excluded from the analyses due to violation of the inclusion criteria. The interviews were performed in the recruiting centers or at the Institute of Medical Psychology and Medical Sociology of the University Medical Center Goettingen. Each interview consisted of two parts and was conducted alike within the different age groups (5–7, 8–12, 13–17), with either C&A or with their parents encouraging a free discussion. The sessions were conducted by a main and an assistant moderator and took around 60 minutes. In the first part, an interview guideline was used covering questions about happiness, wellbeing, satisfaction, limitations, problems, disturbances, friends and family, kindergarten and school, changes in life after TBI, as well as medical and psychological care.

In the second part, participants were presented adapted versions of the QOLIBRI for adults [17, 18] adjusted for each age group. Items were inspected concerning their wording, comprehensibility and relevance, and the suitability of different answer formats. This took around 30 minutes.

Eight interviewers (six female and two male) collected data through semi-structured interviews. The moderators of the interviews held at least a bachelor degree in psychology. Each moderator was trained to use the technique of semi-structured interviews based on an interview guideline. The only previous contact to the interviewed parents was when organizing the interview date per telephone. Details of individual cases were not known to the moderators prior to the interviews. All participants were interviewed face to face in a quiet room at the recruiting center.

## Data analysis

All interviews were recorded, transcribed verbatim, and analyzed according to Mayring [38] with MAXQDA (v.12).

To generate material for later the item formulation and to identify relevant HRQoL dimensions for C&A after TBI, the contents of the transcribed interviews were analyzed by three independent raters in an iterative process. Categories were obtained using a combined approach of deductive and inductive derivation. During the process of content analyses of the statements, a coding guide was developed including definitions and anchor examples for each category as well as general coding rules. After all the interviews had been analyzed, categories and subcategories were assigned to dimensions of HRQoL according to the HRQoL model by von Steinbuechel et al. [17, 18]. Additionally, the category “Covariates” HRQoL was created. Finally, three raters carried out an independent consistency check of the coded statements. Initial agreement between the raters was 84.4%. All disagreements were then discussed until a consensus was reached.

### Results

**Fig 1:**
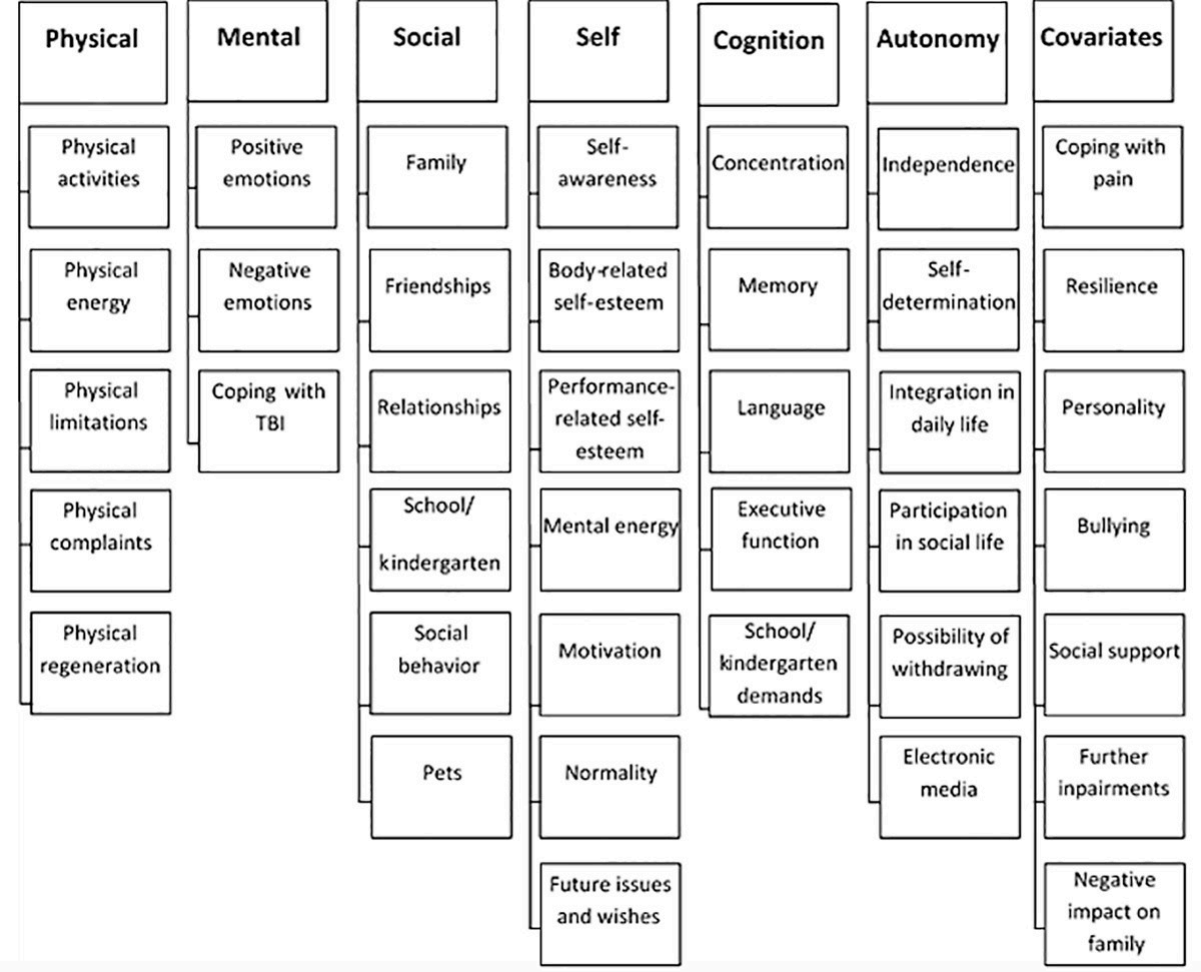
Category system.

Information of 19 C&A and 26 parents was included in the content analyses. Of those, 17 were children-parent pairs. Two participants were interviewed in the absence of their parents, and three parents participated in the interview without their children. To determine whether data obtained in the individual interviews the single child (19 statements, M = 13.6, 95% CI [2.73, 24.47]) and its parent (15 statements, M = 14.1, 95% CI [6.45, 21.88]) could be included in the analyses, the number of coded statements was compared with the mean value of the coded statements of the corresponding age groups. The number of statements in both cases was in the middle of the group ranges covering a broad spectrum of contents. Table 1 provides demographic—information on the participants. The resulting category system regarding dimensions of HRQoL of C&A after TBI is shown in Fig 1.

**Table 1:** Demographic characteristics of participants (N = 45).

		**Frequency**
**Children& Adolescents**		
**Gender**	*Female*	13
	*Male*	6
**Age group (years)**	*5–7*	4
	*8–12*	6
	*13–17*	9
	*Mean (SD)*	11.32 (3.43)
**TBI-severity**	*I*	12
	*II*	4
	*III*	3
**Parents**		
**Gender**	*Female*	18
	*Male*	8
**Child/Parent pairs**	*17 matched*	*2 C&A*, *3 parents non matched*

**Fig 2:**
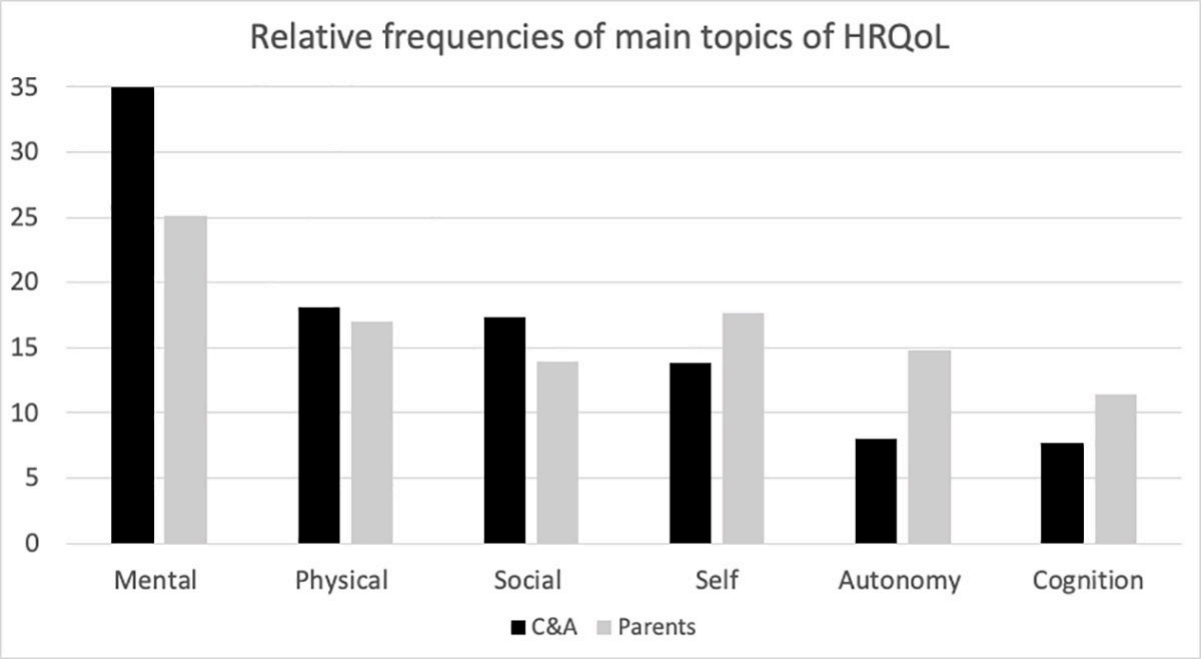
Relative frequencies of topics in statements for children and their parents.

Overall, 375 statements of C&A and 323 their parents were coded in the main categories. Fig 2 shows the relative frequencies of topics mentioned during the interviews reported separately for C&A and their parents.

The frequency of statements coded under the main categories shows a tendency towards similarities between C&A and parents; differences in content between C&A and their parents were found within the subcategories. The statements and topics presented in the following sections may serve as examples of the most relevant issues reported by C&A and their parents. Further reported analyses of the parent-child comparison of statements will categorize them under the main dimensions of HRQoL as these may be especially relevant for item generation.

### Mental

Regarding emotions and feelings, C&A reported positive emotions especially with respect to meeting friends, spending time with their families as well as with their animals, and doing sports. Parents, on the other hand, believed their children to be happy when engaging in computer games, social media or music as well as when spending time with others. In addition, C&A were happy when they had the opportunity to have new experiences (e.g. “[…] that you just live every day and can always make something new out of it.”). Regarding negative emotions, both, C&A and their parents, talked about sadness, injustice, stress, shame, boredom, anxiety and loneliness. Coping with the event leading to TBI was an important issue in the C&A group (e.g. “[…] whenever I remember that moment, I would like to start crying, no idea why.”). Avoidance behavior was another relevant subject of discussion in both groups (e.g. “since the accident, I haven’t played football or similar games anymore, because I don’t like playing games that involve balls anymore.”). In addition, with respect to coping with TBI, parents mentioned cautious behavior of C&A since TBI (e.g. “when she accidently hit herself, she wants to go straight to the hospital.”).

### Physical

This category focuses on physical condition and physical functioning. Here, C&A and their parents emphasized the importance of sport activities. In contrast to their parents, for whom sports predominantly means being physically active, for C&A sport also means spending time with friends. Fatigue was another important issue for both C&A and their parents, with parents emphasizing the importance of taking enough breaks. In addition, C&A focused on physical limitations and experienced unhappiness when they were excluded from sports. Another topic mentioned by C&A was the need to exercise when feeling the urge to move. When C&A and parents talked about pain, they often reported frequently recurring headaches, which they related to stress and efforts at school (e.g. "I have never had such a bad headache before and I’d like to say that when I was in school, the first two lessons were okay and in the third/fourth lessons I always felt going to bed."). Regarding physical regeneration, C&A were happy that many of the physical complaints due to their TBI had subsided by the time of the interview. In addition to physical regeneration, parents reported about the benefits of rehabilitation facilities and physiotherapy.

### Social

Here the focus lies on the interactions of C&A with others. Most of the statements by C&A concerned family and friendship. They talked about missing their families, when they are alone and that the family plays a central role in their life. In contrast, parents noted how important friendships are to C&A and saw themselves in a secondary position. According to the parents, C&A like to have their families close to them. In addition, when talking about leisure activities, friendships are highly relevant to C&A. Animals were regarded as important by both, the children and their parents whether in terms of spending leisure time with them or simply expressing a desire to have a pet of their own. Kindergarten and school were issues often associated with friends (e.g. “I get along well at school, because I have lot of friends.”). Parents considered school and kindergarten to be a pleasant place for C&A but put less emphasis on social interaction compared with C&A. The social behaviors mentioned by parents had negative connotations: social withdrawal, outbursts of rage, as well as problematic behavior of their children (e.g. “Aggression has definitely increased in him.”). In this context, some of the C&A, also reported social withdrawal at school (e.g. “I always distance myself from the people at school.”). In contrast to the parents, C&A also noted positive changes in their behavior (e.g. “So the accident was not so good, but it has done something with me, because before my accident, I was very antisocial to my fellow humans.”).

### Self

This dimension is mainly characterized by the perception of self, and by satisfaction and difficulties with oneself. For C&A and their parents the main topic related to self was physical self-esteem. Both groups emphasized the presence of scars as a result of the accident (e.g. “[…] this (scar) runs from top to bottom, which bothers me.”; “Mom, please give me something long to wear, so no one can see the scars.”). Some C&A wished for more self-confidence (e.g. “I am not the same anymore, my self-esteem has dropped a bit.”). Regarding performance-related self-esteem, both C&A and their parents stressed the children’s academic and athletic achievements since the accident (e.g. “I am actually quite good at school”). Furthermore, C&A reported an important desire to be treated normally, and not to be overprotected or favored because of the TBI (e.g. “I do not want the teachers to be so considerate–it has already been a year, I feel better now and I would like to graduate and be able to do it alone.”). Parents also recognized the desire of C&A to be treated normally, asking themselves what they could improve and how they could encourage their children (e.g. “[…] of course, we try to convince her that she is something special.”). Additionally, parents considered mental energy to be another important issue (e.g. “At school, he gives 100%, but an average person of his age just needs 80% to get the same performance—it depends on his fatigue.”). Finally, for C&A and their parents, future aspects and wishes included career plans, wishes for objects (e.g. laptop) and family happiness.

### Cognition

We also investigated whether TBI had an impact on the perception and evaluation of C&A’s ability to think. The demands imposed by schools and kindergartens were the main issues for C&A and their parents with respect to cognitive skills (e.g. “I always had difficulties in geometry, but after TBI it became worse.”; “In terms of school performance, he was like on a zero position—he could not get the ABC, 123, nothing…”). Parents and C&A, especially adolescents, discussed the pressure to perform and being able to keep up with others cognitively. Concentration and attention problems were also intensely debated by both groups (e.g. “Even before the accident my concentration was not too good, but it got worse.”; “She had no idea of English grammar, nor the vocabulary she had learned years ago—it was completely gone.”). Both, C&A and their parents, mentioned orientation and memory problems (e.g. “I had problems with my short-term memory, because if someone told me something, 10 minutes later I did not know anything about it.”). In contrast to C&A, parents emphasized language issues. Here semantic and phonetic paraphasia, syntax problems as well as slow speech were discussed. In addition, parents talked about reading difficulties, changes in fine motor skills and focused attention (e.g. “She will be doing math, but then she completely turns away and starts telling me something else that happened a week ago or whenever.”).

### Autonomy and daily life

This category of HRQoL is associated with the ability to deal with things alone and to manage everyday activities. The issue of independence, managing things on their own, as well as dealing with their own insecurities, were important to C&A. In contrast to this, parents reported that C&A wish to have them nearby (e.g. “[…] He wants to become a bit independent, but not really […] with an eye on whether anyone is there.”). Self-determination was an important issue in C&A’s everyday lives (e.g. “[…] that you have many opportunities to do things you want to, that you can travel, meet friends—you can do so much.”). Likewise, their parents addressed the importance of freedom to them. The possibility of withdrawing means a lot to C&A (e.g. “Sometimes, during bad times, I have to cry, but then I say: please, leave me alone, I will come to you soon.”); this was also mentioned by their parents. According to C&A and their parents, integration into daily life through an adaptation phase is helpful. Additionally, parents emphasized the importance of supporting their children and the possibility of their children being able to return to the same class they were in before TBI (e.g. “The most important thing is to return him to his social environment and then we will see how the teachers and classmates handle it.”). Participating in social life, i.e. being part of an association, is important to both groups, with parents’ putting less value on it than C&A. Electronic media, such as watching series, playing on their mobile phones, are further important contents of everyday life for C&A, also from their parents’ perspective.

### Analysis of age-specific aspects of HRQoL

**Fig 3:**
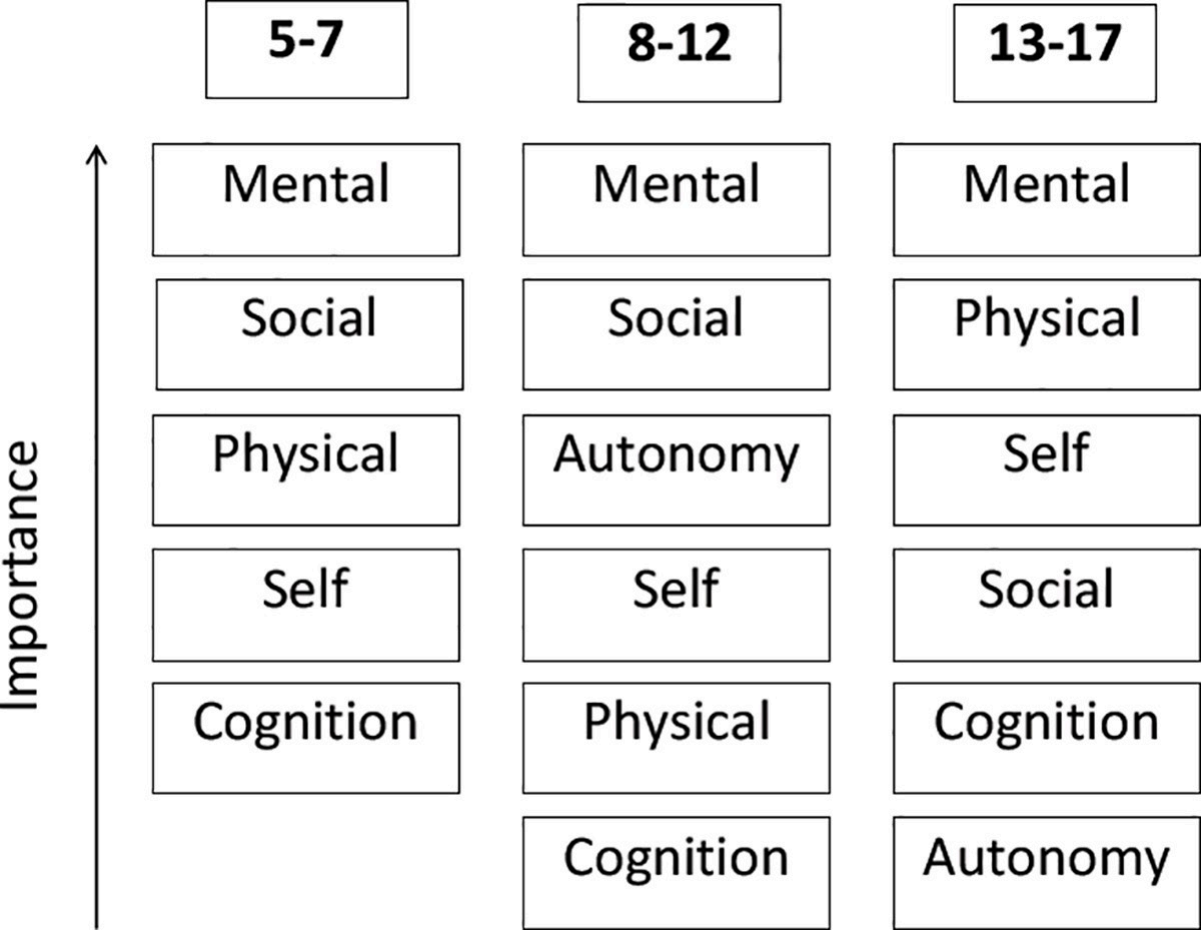
Main age-related categories of HRQoL for children and adolescents.

**Fig 4:**
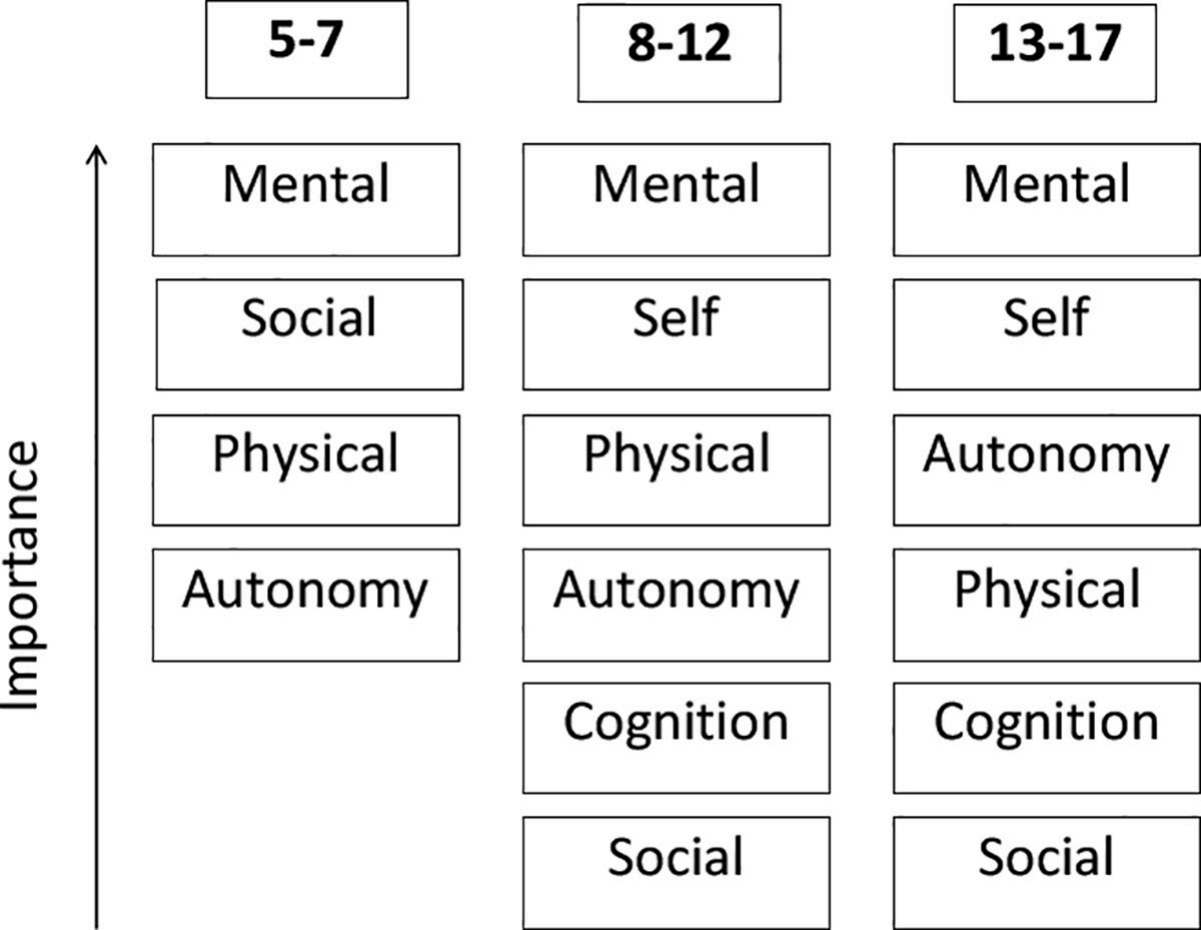
Main age-related categories of HRQoL for parents.

We also investigated age-specific aspects of the main categories of HRQoL by analyzing differences in the relevance of topics for C&A depending on age. Fig 3 depicts the categories which were intensively discussed by the C&A in each age group. Fig 4 shows the main topics mentioned by the parents.

The results reveal age-specific differences between C&A and their parents regarding the frequency of the named categories. Only three matching categories emerge for all age groups, namely mental, social and physical HRQoL. While children between the ages of five and seven years focused on the topics of cognition and self, their parents mainly discussed autonomy and daily life. These topics, on the other hand, were mentioned by C&A in the 8–12 age group. In addition, the order of importance differed between C&A and their parents in all age groups.

## Discussion

The interviews conducted with C&A and their parents in this study provided valuable material to generate items for the development of TBI-specific and age-adapted questionnaires for C&A after TBI and their parents to assess disease-specific HRQoL. Results show that C&A and parents largely discussed the same main topics, implying that parents may be able to recognize what is relevant and important to C&A after TBI. These results are to some extent contrary to the findings of Erickson et al. [39], who reported, that children and parents perceive HRQoL differently. Also, in our study, C&A and their parents prioritized different aspects, especially in subcategories such as coping with TBI, physical regeneration, social behavior, independence, language, concentration and mental energy. In accordance with the findings of Limond et al. [40], parents more frequently than C&A mentioned behavioral problems. Overall, they pay more attention to limitations. Regarding age relevant aspects, differences between C&A and their parents were found: parents of the younger age groups focused on topics such as autonomy before these became relevant to the C&A themselves. This is in line with the results of Upton et al. [27] and Davis et al. [41] who identified differences in the agreement of HRQoL domains between C&A and parents. Also, Green and colleagues [42] found an acceptable conformity for psychosocial outcome between parents’ and adolescents’ ratings, although there were discrepancies in HRQoL ratings. Therefore, under circumstances in which C&A after TBI are not able to give reliable information about their HRQoL themselves, a proxy assessment might serve as a surrogate, but possible biases need to be critically evaluated.

TBI affects various areas of life, for example in the field of cognition, such as memory disorders, attention problems, or physical limitations and pain. These in turn can negatively affect self-esteem, social and family networks. Predominantly through group interviews with C&A after TBI and their parents’ specific topics and important aspects of their HRQoL were identified leading to the wording of the items for the QOLIBRI-Kiddy/Kid/Ado and proxy questionnaires to be tested in a further study. A TBI-specific HRQoL instrument could help to determine those aspects that are significant for the HRQoL of C&A after TBI and improve medical and psychological care. The results of this qualitative study confirm the necessity to involve those affected in HRQoL instrument development. The inclusion of young children, especially via self-report, has previously been recommended by other researchers [43].

The results of this qualitative study were obtained with rather small samples and are therefore only exploratory. Relevant information concerning the content and formulation of possible items was determined allowing to develop age-adapted instruments assessing HRQoL after pediatric TBI in C&A and their parents or proxies. A generalization of these findings will be possible after the finalization of the psychometric testing and validation study of the different questionnaires. Age-adapted instruments [17, 18], will allow to monitor long-term follow up of HRQoL after TBI from childhood to old age with the same instrument modules. This is of great importance, as TBI may affect the lives of patients and their families for their entire lives [44–47].
